# Characterization of human enterovirus71 virus-like particles used for vaccine antigens

**DOI:** 10.1371/journal.pone.0181182

**Published:** 2017-07-21

**Authors:** Dandan Zhao, Bo Sun, Shiyang Sun, Bin Fu, Chuntian Liu, Dawei Liu, Yanfei Chu, Youlei Ma, Lu Bai, Yongge Wu, Yan Zhou, Weiheng Su, Ali Hou, Linjun Cai, Fei Xu, Wei Kong, Chunlai Jiang

**Affiliations:** 1 National Engineering Laboratory for AIDS Vaccine, School of Life Sciences, Jilin University, Changchun, China; 2 School of Life Sciences, Jilin Agricultural University, Changchun, China; 3 Beijing Proteome Research Center, Beijing, China; 4 Changchun BCHT Biotechnology Company, Changchun, China; Sun Yat-Sen University, CHINA

## Abstract

Human enterovirus 71 (EV71) is a major causative pathogen of hand, foot and mouth disease (HFMD) and has caused outbreaks with significant mortality among young children in the Asia-Pacific region in recent years. Towards developing a vaccine for this disease, we have expressed and purified EV71 virus-like particles (VLPs), which resemble the authentic virus in appearance, capsid structure and protein sequence, from insect cells (Sf9) using a multistep chromatography process. We demonstrated intracellular localization of the VLPs in host cells by *in situ* immunogold detection, electron microscopy and immunofluorescence. Characteristics of these EV71 VLPs were studied using a variety of immunological and physicochemical techniques, which aimed to reveal that the purified EV71 VLPs have good morphology and structure consistent with natural EV71 empty capsids. Results of the amino acid analysis, SDS-PAGE, Western blotting and high-performance liquid chromatography confirmed the high purity of the EV71 VLPs. However the sedimentation coefficient of the VLPs showed that they were smaller than that of secreted EV71 VLPs purified by discontinuous cesium chloride density gradients, they were similar to the empty capsids of natural EV71 virions reported previously. Combined with the previous study that EV71 VLPs purified by a multistep chromatography process were able to elicit strong humoral immune responses in mice, our results further supported the conclusion that our EV71 VLPs had well-preserved molecular and structural characteristics. The EV71 VLPs produced from the baculovirus expression system and purified by a multistep chromatography process displayed key structural and immunological features, which would contribute to their efficacy as a HFMD vaccine.

## Introduction

Hand, foot and mouth disease (HFMD) is an infectious disease found in infants and young children worldwide and has become a major public health concern across the Asia-Pacific region since 1997. Although this illness is mild and self-limiting in most instances, cases caused by human enterovirus 71 (EV71) can result in severe neurological complications and even death among children under five years of age [1–6]. In mainland China, cumulatively 16,291,933 HFMD cases including 3515 deaths have been reported from 2008 to 2016 (www.chinacdc.cn). Human EV71, which is a non-enveloped, single positive-stranded RNA virus that is a member of the *Enterovirus* genus in the *Picornaviridae* family, has been identified as the major causative agent [7–9]. Therefore, a more effective vaccine against EV71 for controlling and preventing HFMD which is highly desirable.

Various vaccine candidates against EV71 virus, including inactivated vaccines [10, 11], live attenuated vaccines [12], virus-like particle (VLP) vaccines [13, 14], subunit vaccines based on the VP1 protein [15] and epitope-based vaccines [16, 17], have shown different levels of efficacy in animal studies or human clinical trials. However, most studies aimed at preventing HFMD have mainly focused on inactivated virus vaccines. In Singapore and Taiwan, inactivated vaccines based on genotype B3 and B4, respectively, have completed phase I clinical trials. In mainland China, three inactivated vaccine candidates based on genotype C4 have completed testing in phase III clinical trials [10, 11], and one of the inactivated vaccine candidates has been on the market in 2016. However, the precise safety and efficacy profiles of these vaccines remain to be further identified whether they can be used widely in the target population.

There are many VLPs of different viruses which have been produced and studied as candidate vaccines. Many VLP-based vaccines, such as influenza (Novavax), hepatitis B virus (Merck) and human papillomavirus (Merck) have been licensed. Given that VLPs resemble authentic virions in terms of their structural proteins and lack of a viral genome, they have been as promising candidates for vaccine development. Chung et al. [18, 19] have produced and evaluated the EV71 VLPs generated in the baculovirus-insect cell expression system, and these EV71 VLPs purified by discontinuous CsCl gradient have been demonstrated to protect newborn mice against EV71 lethal challenge. Although the EV71 VLPs as a vaccine is promising, the high purity and quantity of EV71 VLPs, which can be used in vaccine production, should be further studied and tested. Our previous study showed that a VLP expression system based on a baculovirus (Bac-P1-3CD) co-expressing EV71 structural protein P1 and 3CD protease in Sf9 cells have been established, which could self-assemble into VLPs [20]. The EV71 VLPs were purified via a novel multistep chromatography process using Capto^TM^ Core 700, Capto^TM^ Adhere resin and Capto^TM^ Butyl (GE Healthcare, Piscataway, NJ, USA) columns, resulting in VLPs with ~31.52% yield and of > 95% purity [21]. We also showed that the EV71 VLPs could induce a high titer of neutralizing antibodies, which can protect newborn mice from lethal challenge with the EV71 C4 strain [22]. How the EV71 VLPs vaccine mimics its viral counterpart molecularly and structurally is an issue of great interest for effective vaccine development. In this study, we report that EV71 VLPs, produced by baculovirus expression vector system (BEVS), mainly localized in the cytoplasm where they self-assembled to form VLPs. The characteristics of the EV71 VLPs were studied using many kinds of physicochemical and immunological techniques. Determination of the sedimentation coefficient of EV71 VLPs using analytical ultracentrifugation (AUC) indicated that they were smaller than the secreted EV71 VLPs purified by discontinuous cesium chloride density gradients but were similar to the empty capsids of natural EV71 virions reported previously [23, 24]. Altogether, this and previous studies not only show that the obtained EV71 VLPs were immunogenic and could induce neutralizing antibodies by immunization in mice [22], but they also provide a structural basis for the generation of VLP vaccines produced from Sf9 cells and purified by a novel multistep chromatography process. Such findings facilitate the potential development of an EV71 VLPs vaccine candidate against HFMD.

## Materials and methods

### Expression and purification of EV71 VLPs

The Sf9 insect cells derived from ATCC CRL-1711 were cultured in 2 L of Serum-Free Insect Cell Culture Medium (SFX) (GE Healthcare, Piscataway, NJ, USA), which were infected with a recombinant baculovirus (Bac-P1-3CD) constructed using the Bac-to-Bac^TM^ system (Invitrogen, Carlsbad, CA, USA) to co-express P1 and 3CD based on a Chinese endemic genotype C4 strain EV71, AH08/08 (GenBank no. HQ611148) to produce EV71 VLPs, as described previously [21]. The production of EV71 VLPs cultivated in a WAVE bioreactor (GE Healthcare, Piscataway, NJ, USA) was described in detail previously. Sf9 cells pelleted from a 2 L culture were resuspended in 220 mL phosphate-buffered solution (PBS, pH 7.4) followed by sonication at 4°C with 40% of max power 750 W to release the intracellular EV71 VLPs. The cellular debris was pelleted through centrifugation at 16,000 × *g* for 30 min at 4°C, and the supernatant was obtained with a 0.45-μm cut-off filter (50 mm diameter; Millipore, Darmstadt, Germany) and then loaded onto chromatography columns for purification. A novel multistep chromatography process using Capto^TM^ Core 700, Capto^TM^ Adhere resin and Capto^TM^ Butyl (GE Healthcare, Piscataway, NJ, USA) columns was used, resulting in EV71 VLPs with ~31.52% yield and of > 95% purity as previously described [21].

### Mass spectrometry analysis of EV71 VLPs

For the peptide mapping and N- and C-terminus sequencing by liquid chromatography-mass spectrometry, the purified EV71 VLPs samples were reduced with 10 mM dithiothretiol (DTT) in 100 mM NH_4_HCO_3_, alkylated with 50 mM indoleacetic acid (IAA) in 100 mM NH_4_HCO_3_ and divided into three parts. The three samples were digested for 12–16 h at 37°C with a solution of trypsin, chymotrypsin or endoproteinase Glu-C (protease-to-substrate ratio was 1:50). To identify the N-glycosylation sites of EV71 VLPs, a solution of PNGase-F (protease-to-substrate ratio was 1:20) was adopted to digest the purified VLPs samples at 37°C for 12–16 h. Thereafter, the molecular weights of the peptide fragments and their amino acid sequences were measured by tandem mass spectrometry (LC-MS/MS) by using Q Exactive (Thermo Fisher Scientific, Waltham, MA, USA). The HPLC system consisted of an Eksigent Nano2D LC (Eksigent Technologies, Silicon Valley, CA, USA) and an Acclaim^®^ PepMap300 C18 (15 cm long, 75 μm inner diameter, 5 μm, 300Å) (Dionex, Sunnyvale, CA, USA). Electrospray MS/MS spectra were allocated to the EV71 VLPs primary sequence using Mascot 2.1.0 (Matrix Science, London, UK) software, and an in-house protein sequence database was established. The mass tolerance on the parent and the fragment ions were 0.1 Da and 0.2 Da, respectively. The variable modifications allowed were carbamidomethyl (C), oxidation (M) and deamination (D and E). The N-glycosylation sites were determined by the quality drift (0.984 Da) of asparagine changed to aspartic acid [25].

To identify the molecular weights of VP0, VP1 and VP3 capsid proteins of EV71 VLPs, the purified EV71 VLPs were mixed (1:1) with 5 mg/mL alpha-cyano-4-hydroxy-cinnamic acid (CHCA) containing 0.1% trifluoroacetic acid (TFA) and 50% acetonitrile. After mixing, 0.8 μL of the mixture was used for MS analysis, and then a MALDI-TOF/TOF mass spectrometer 4800 Proteomics Analyzer (Applied Biosystems, Framingham, MA, USA) was adopted. Data Explorer (TM) software (Applied Biosystems) was used to analyze the data.

### Immunofluorescence staining and confocal microscopy

The Sf9 cells were seeded (5 × 10^5^ cells per well) onto 24-well plates 1 day prior to the experiment. On the second day, recombinant baculovirus Bac-P1-3CD diluted in SFX medium was added to each well at 1 multiplicity of infection (MOI), followed by incubation at 27°C for 24 h, 48 h or 72 h to observe the production of EV71 VLPs. Uninfected Sf9 cells were used as the blank control. Thereafter, the cells were prepared for immunostaining. In brief, 4% paraformaldehyde was used to fix cells at ambient temperature for 20 min, and then followed by treatment with permeabilization buffer (0.1%Triton X-100 in PBS) for 30 min. After cell fixation, the cells were blocked with PBS containing 5% bovine serum albumin (BSA) for 1 h and then incubated with the 1/50-diluted anti-VP1 rabbit polyclonal antibody for 1 h. Infected Sf9 cells incubated with the 1/50-diluted serum from rabbits immunized with PBS were used as the negative control to monitor non-specific staining. Next, the cells were incubated with Alexa Fluor 488-conjugated anti-rabbit IgG (Life Technologies, Carlsbad, CA, USA) for 45 min. The PKH26 Red Fluorescent Cell Linker Kit (Sigma, St. Louis, MO, USA) was used as the standard protocol to label the lipid bilayer of the cell membrane, followed by 4',6-diamidino-2-phenylindole (DAPI) (Beijing Biosynthesis Biotechnology Co., Ltd., Beijing, China) staining for 10 min. All incubation steps were carried out at 27°C, and the cells were washed thrice with PBS between steps. Finally, a Zeiss LSM 710 laser scanning confocal microscope (Zeiss, Germany) was used to test the stained samples at 400× magnification, and the Zeiss Zen Lite software was adopted to collect and analyze images.

### Cellular expression and immunolocalization by ultrastructural electron microscopy

Pellets of EV71 VLP-producing cell cultures infected for 72 h were resuspended after centrifugation, and were then washed thrice with PBS before fixing for 2 h at room temperature in 4% paraformaldehyde. To remove the fixative agent, we washed the cell suspension thrice with PBS. The cells were resuspended in PBS rapidly and then centrifuged for 2 min at ambient temperature. Small blocks (<1 mm^3^) were then cut and washed, and LR White Resin (SPI, Berkshire, UK) was used to infiltrate the blocks for embedding at 4°C. The steps were: dehydration at 4°C in 50% ethanol (20 min); at 4°C in 70% ethanol (2 × 30 min) followed by a 1:2 mixture of 70% ethanol and LR White (60 min), LR White (60 min); then two changes of pure resin (1 h, and overnight). After these processes, the blocks were first transferred into fresh LR White with LR White accelerator (2 μL/mL) capsules and then polymerized at 50°C for 24 h, which were trimmed to cut into 70 nm-thick sections using the LEICA EM UC7 ultramicrotome. Sections were mounted on formvar that was coated nickel grids (Gilder 200 mesh), and were then rehydrated by floating the grid on a droplet (20 μL) of PBS contained BSA (1%, w/v) for 30 min at ambient temperature. A droplet of anti-VP1 rabbit polyclonal antibody, prepared in-house as previously reported [21] and diluted 50-fold in PBS + 0.1% BSA, was transferred to the grid and incubated for 1.5 h at ambient temperature. The grid was incubated for 45 min at ambient temperature with goat anti-rabbit-gold complexes (15 nm ∅; 1:50 v/v; Beijing Biosynthesis Biotechnology Co., Ltd., Beijing, China) in PBS + 0.1% BSA after it was washed with PBS + 0.1% BSA (3 × 5 min). The grid was ultimately washed with PBS and distilled water in turn before being dried thoroughly. The material was stained with uranyl acetate and observed under an H-7650 (Hitachi, Tokyo, Japan) transmission electron microscope at 80 kV.

### SDS-PAGE, Western blotting and high-performance liquid chromatography (HPLC) analysis

Purified EV71 VLPs were first treated with reducing SDS-PAGE sample buffer (pH 6.8) and run through a 13.5% separating gel. After the run, the electrophoretic profiles were revealed by silver staining with standard protocols. A Bio-Rad model 583 gel dryer (Hercules, CA, USA) was used to dry the gels. Proteins (3 μg/well) were loaded for silver staining.

After electrophoresis, the purified EV71 VLPs were then transferred onto nitrocellulose membranes (Bio-Rad) for Western blotting, and the membranes were incubated with an anti-VP1 rabbit polyclonal antibody, anti-VP2 mouse polyclonal antibody detecting VP0 protein or anti-VP3 mouse polyclonal antibody (prepared and supplied by our laboratory) for 90 min at ambient temperature. After incubating with the primary antibody, the membranes were incubated with the appropriate alkaline phosphatase (AP)-labeled secondary antibody (Jackson Immuno Research, West Grove, PA, USA) at ambient temperature for 45 min. The AP substrate solution of nitroblue tetrazolium (NBT) and 5-bromo-4-chloro-3-indolylphosphate (BCIP) (Sigma) was used to incubate at ambient temperature for 5 min, and distilled water was finally adopted to wash the membrane. The presence of VP0, VP1 and VP3 capsid proteins of EV71 VLPs in the purified sample was confirmed.

For HPLC, as our previous study the HPLC TSK gel® G5000 (TOSOH, Japan) column was applied to determine the purity of our EV71 VLPs samples [21], which was equilibrated 2 column volumes of 20 mM Na_2_HP0_4_ containing 100 mM NaCl (pH 7.4) before loading 50 μL of our final sample at 15 mL/h.

### Transmission electron micrograph (TEM) and in-solution atomic force microscopy (AFM)

To observe the morphology of VLPs, the EV71 VLPs in suspension were examined using an H-7650 (Hitachi) transmission electron microscope at 80 kV, the specific procedure was detailed as our previous study [21].

The morphology of EV71 VLPs were studied by AFM. Following a published protocol [26], 0.01% (weight/volume) poly-L-lysine solution was coated on mica for 30 min at ambient temperature before the mica was rinsed with Milli-Q water. A 20 μL aliquot of EV71 VLPs (0.3 mg/mL) was applied to a mica surface (1.5 centimeter diameter) at ambient temperature to allow passive adsorption of VLPs from the solution. Without allowing the sample to dry, the mica surface was gently rinsed with PBS. After being dried by nitrogen, the EV71 VLPs were analyzed by AFM. AFM images were collected in the tapping mode in air using a NanoWizard II BioAFM (JPK Instruments AG, Berlin, Germany). Measurements were taken at room temperature (~22°C) using silicon probes (OTESPA-R3, 300 KHz, 26 N/m, Bruker AXS, Germany).

### Dynamic Light Scattering (DLS)

A Zetasizer Nano ZS90 instrument (Malvern Instruments Ltd., Worcestershire, UK) was adopted to determine the hydrodynamic diameter of EV71 VLPs. Measurements were carried out at 25°C using three 10 × 10-sec datasets. All data here are averages of three measurements of the same sample [21].

### Cryo-TEM

For cryo-TEM imaging of EV71 VLPs, a 3.5 μL aliquot of EV71 VLPs (1.5 mg/mL) was applied to glow-discharged Quantifoil R2/2 (Quantifoil Micro Tools GmbH, Jena, Germany), blotted for 2 s in a 100% humidity chamber under 0 blot force at 22°C and then plunged into liquid ethane in an FEI Vitrobot Mark IV vitrification robot. EV71 VLPs were imaged in an FEI 200-kV TECNAI SPIRIT transmission-electron microscope.

### AUC

A Beckman Coulter Proteome Lab XL-I analytical ultracentrifuge with a 4-hole An-60Ti rotor was used to determine the sedimentation coefficient distribution of EV71 VLPs (0.3 mg/mL) contained in 0.1 M PBS, and a continuous scan mode was adopted to measure the sedimentation at 15,000 rpm in 12-mm double-sector cells at 20°C (measured cell radius from 5.9 cm to 7.15 cm), the absorbance at 280 nm. The data in c(s) mode was analyzed by the program Sedfit to show a sedimentation coefficient distribution apparently, and the c(s) calculations were determined by the 3th to 40th scans of EV71 VLPs.

## Results

### Mass spectrometry analysis of EV71 VLPs

**Fig 1:**
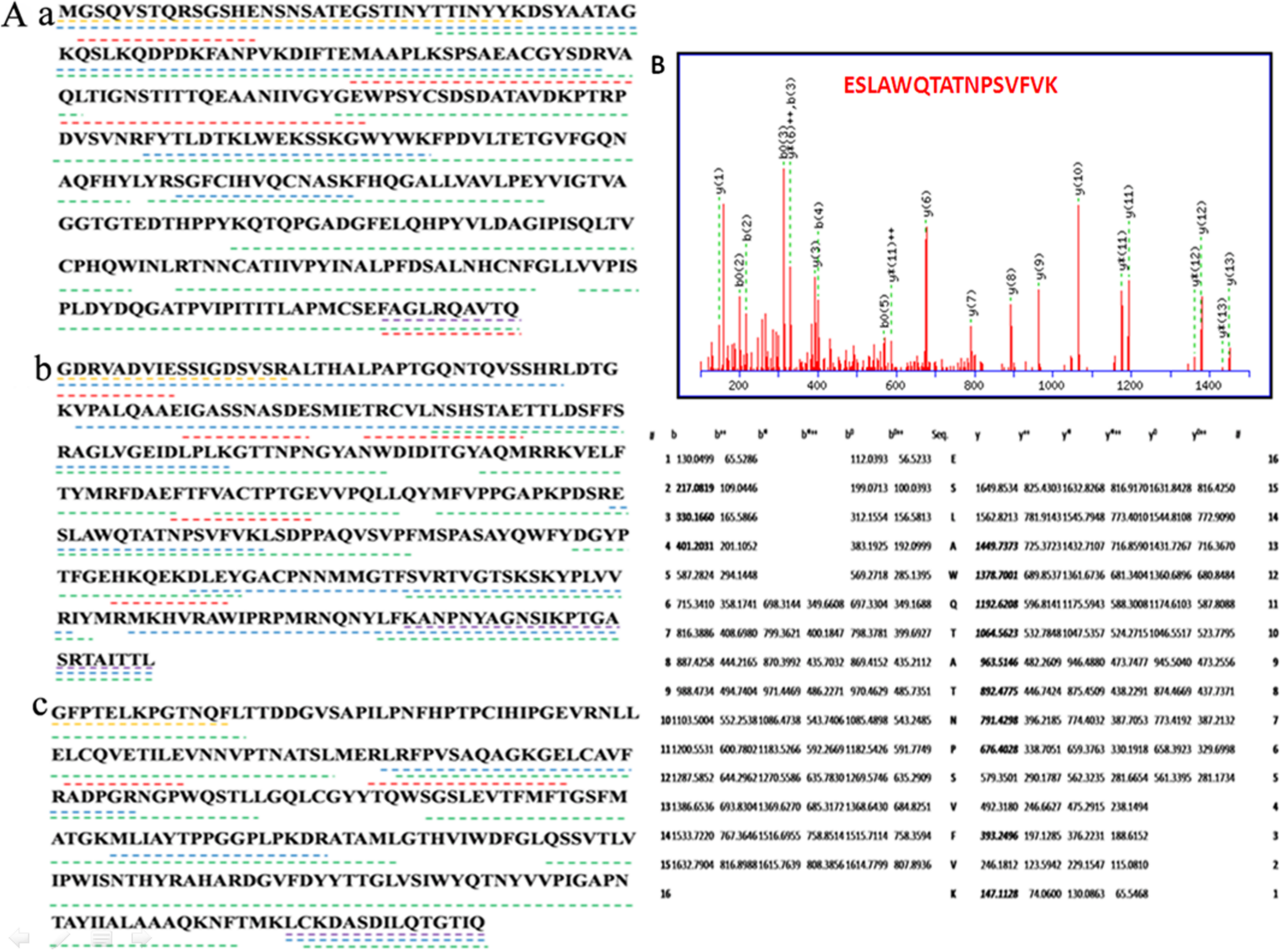
Confirmation of amino acid sequence of EV71 VLPs. Amino acid sequence of VP0 (a), VP1 (b) and VP3 (c) of EV71 VLPs (A), as determined by translation of the corresponding DNA sequences. Peptides are shown that have been obtained by digestion with trypsin (blue), chymotrypsin (green) or endoproteinase Glu-C (red) and identified by LC-MS/MS. The N-terminal sequences are marked in orange and the C-terminal sequences in purple. (B) N-glycosylation site at VP1 residue N^176^ of EV71 VLPs. Monoisotopic mass of neutral peptide Mr (calc): 1777.8887; fixed modifications: Carbamidomethyl (C) (applied to specified residues or termini only); variable modifications: N10: deamidated (NQ); ions score: 57 Expect: 2.1e-006; matches: 21/162 fragment ions using 39 of the most intense peaks. Electrospray MS/MS spectra were assigned to the EV71 VLPs primary sequence using the Mascot 2.1.0 (Matrix Science, London, UK) software, and an in-house protein sequence database was established.

To verify sequences of the BEVS-produced EV71 VLPs capsid proteins, their amino acid sequence was determined by using Eksigent Nano2D LC and Q Exactive after protein hydrolysis of EV71 VLPs. The Acclaim^®^ PepMap300 C18 column (5 μm, 300 Å) was employed for HPLC. The amino acid sequences of VP0, VP1 and VP3 were gained by translation of the corresponding DNA sequences (Fig 1). More than 90% of the VP0 and VP1 sequences were identified by peptide mapping and mass spectrometry, and more than 76% of the VP3 sequence was confirmed. Moreover, no mutations of the N- and C-terminal amino acids of VP0, VP1 and VP3, as compared with the EV71 C4 strain, were found (Fig 1). Determination of the average molecular mass showed that the purified EV71 VLPs consisted of VP0 (35 KDa), VP1 (33 KDa) and VP3 (26 KDa) capsid proteins. The mass spectrometry is of importance for the structural analysis of protein identification to understand the samples quality information. The protein used for vaccine, which assembled in different biological environments, may result in partial deletion of the N-/C- terminal amino acid sequence or different folding forms or post-translational modifications. The mass spectrometry analysis is one of the most effective tools to assess the N-/C- terminal amino acid sequence and determine the protein molecular mass and detect the post-translational modification site[27, 28]. In order to detect the presence of N-glycosylation sites on the EV71 VLPs produced from Sf9 cells, MALDI-TOF/TOF was used for the analysis based on N-linked glycans removed by the PNGase F chemical method. The analysis led to a newly identified N-glycosylation site in EV71 VLPs at the VP1 residue N^176^ (Fig 1B).

### Detection of expression and intracellular localization of EV71 VLPs

**Fig 2:**
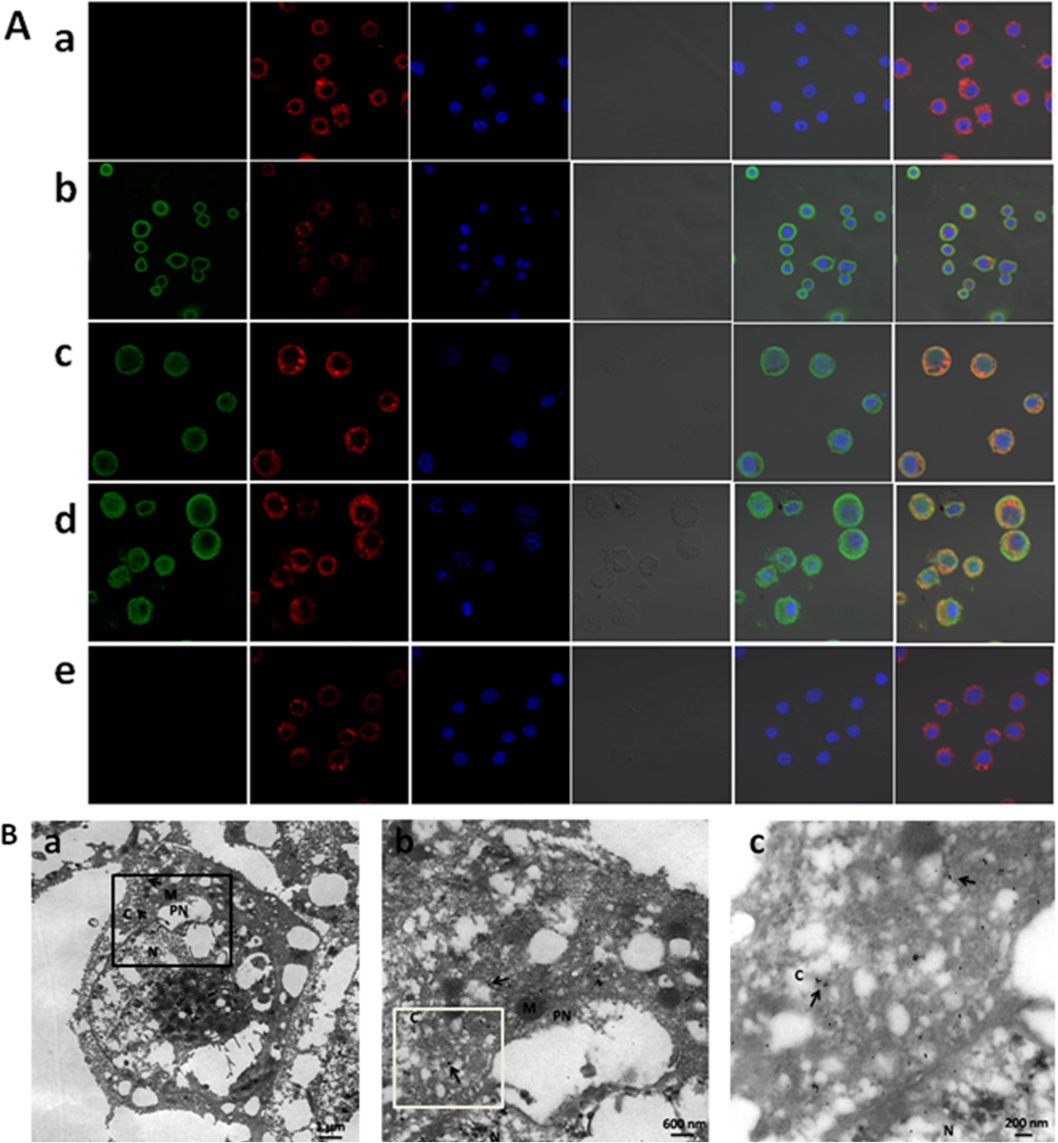
Detection of expression and intracellular localization of EV71 VLPs. (A) Analysis of EV71 VLPs expression by confocal immunofluorescence. Sf9 cells infected with 1 MOI of the recombinant baculovirus Bac-P1-3CD were incubated at 27°C for 24 h (b), 48 h (c) or 72 h (d) in the experimental group. Uninfected Sf9 cells were used as the blank control (a). Serum from rabbits immunized with PBS was used in the negative group (e) to monitor non-specific staining as described in materials and methods. The EV71 VLPs were indirectly stained with anti-VP1 rabbit polyclonal antibody (Alexa Fluor 488, green). The cell membrane lipid bilayer was stained with PKH26 (red). The nuclei were stained with DAPI (blue). Bright field images of cells are shown. Merge 1: green + blue + bright field. Merge 2: green + red + blue + bright field. Images were obtained at a magnification of 400×. NC, negative control; BC, blank control. (B) Immunogold detection with specific polyclonal antibodies against EV71 showing cytoplasmic localization. Sf9 cells were infected with Bac-P1-3CD and prepared for TEM as described in materials and methods. Panel a: TEM showing infected Sf9 cells. Bar: 1 μm. Panel b: A magnified view of the boxed section in panel a. Bar: 600 nm. Panel c: A magnified view of the boxed section in panel b, showing EV71 VLPs labeled by immunogold complexes 15 nm ∅and located in cytoplasm. C, cytoplasm; M, mitochondria; N, nucleus; PN, perinuclear; arrows show localization of gold particles, hence of EV71 VLPs. (a) bar = 1 μm; (b) bar = 600 nm; (c) bar = 200 nm.

In our study, anti-VP1 rabbit polyclonal antibody labeled by green fluorescence dye or gold particles were mainly detected in the cytoplasm of infected Sf9 cells, which indicated that EV71 VLPs were mainly expressed in cytoplasmic locations of the host cells. Thus, VLP formation occurred inside the Sf9 cells when they were infected with 1 MOI of the recombinant baculovirus Bac-P1-3CD. These findings were determined by immunofluorescence and *in situ* immunogold staining of baculovirus-infected cells using anti-VP1 polyclonal rabbit antibodies specific to EV71 VLPs (Fig 2). Compared with uninfected cells, the infected cells grew larger in diameter with time and showed the highest amount of EV71 VLPs expression at 72 h, as shown by immunofluorescence analysis (Fig 2A). Hence, the infected Sf9 cells were harvested during that time. These findings are similar to observations in a previous report [29]. The membrane structure in the cytoplasm also appeared to increase with time (Fig 2A), which may be due to the development of apoptosis after infection with the recombinant baculovirus. A major feature of infection by the BEVS is the massive reorganization of nuclei in which they expand to such an extent that they fill most of the cell volume (Fig 2B). At this stage, nucleocapsids of baculovirus are likely destined to develop into budded virus [30], and the EV71 VLPs are then expressed abundantly in low MOI infection cultures.

### Purity analysis of EV71 VLPs

**Fig 3:**
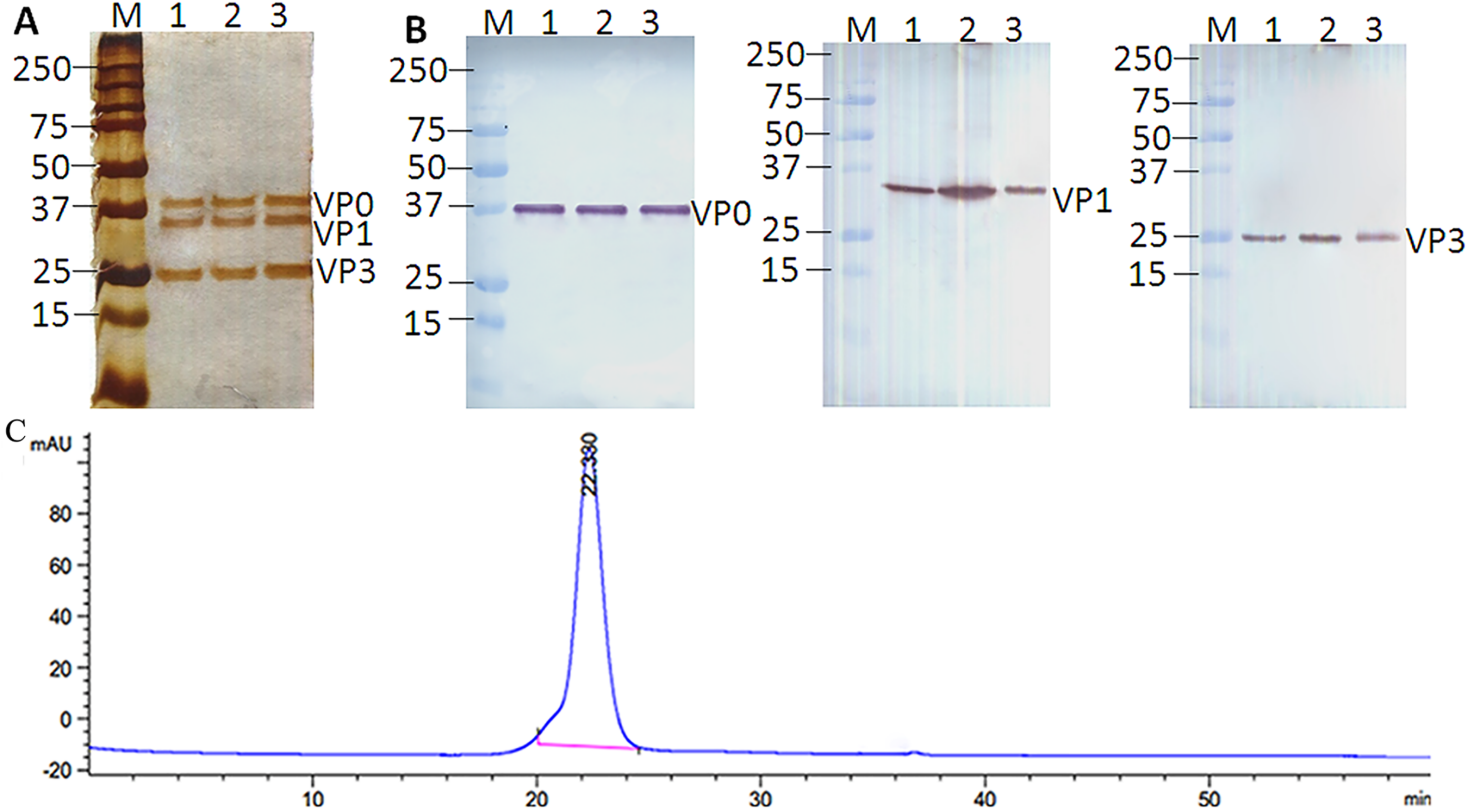
Purity analysis of EV71 VLPs. SDS-PAGE analysis of purified EV71 VLPs (3 μg/well) from three production batches (one batch per lane), stained with silver (A). Alternatively, after SDS-PAGE, proteins were transferred onto blotting membranes and probed with EV71-specific antibodies (B). Anti-VP1 rabbit polyclonal antibody, anti-VP2 mouse polyclonal antibody detecting VP0 protein and anti-VP3 mouse polyclonal antibody were used in the Western blot. (C) EV71 VLPs (50 μL) after final purification were detected by TSK gel® G5000.

In an attempt to produce and purify EV71 VLPs, a Bac-to-Bac^TM^ system co-expressing P1 and 3CD derived from a Chinese endemic genotype C4 strain EV71 was generated as previously reported [20]. The recombinant baculovirus (Bac-P1-3CD) was used to infect Sf9 cells and cultivated in a WAVE bioreactor. The cells were harvested 3 days post-infection and then subjected to a novel multistep chromatography purification process, resulting in EV71 VLPs with ~31.52% yield as previously described [21]. Different batches of EV71 VLPs were analyzed by SDS-PAGE and Western blotting (Fig 3). SDS-PAGE staining with silver (Fig 3A) was adopted to determine the proportion of EV71 VLPs in our purified sample, which showed only three bands with approximate molecular weights of 36, 33 and 25 kDa, corresponding to VP0, VP1 and VP3, respectively, and indicated a purity of the EV71 VLPs that was greater than 95%. This result agrees with that described by Ku *et al*. [31]. These bands were found by further Western blot analysis using corresponding polyclonal antibodies (Fig 3B). SDS-PAGE and Western blot experiments observed for all batches were to indicate that the process can consistently yield highly pure EV71 VLPs, and the HPLC analysis also further confirmed that the purity was above 95% (Fig 3C).

### Morphology and diameter analysis of purified EV71 VLPs

**Fig 4:**
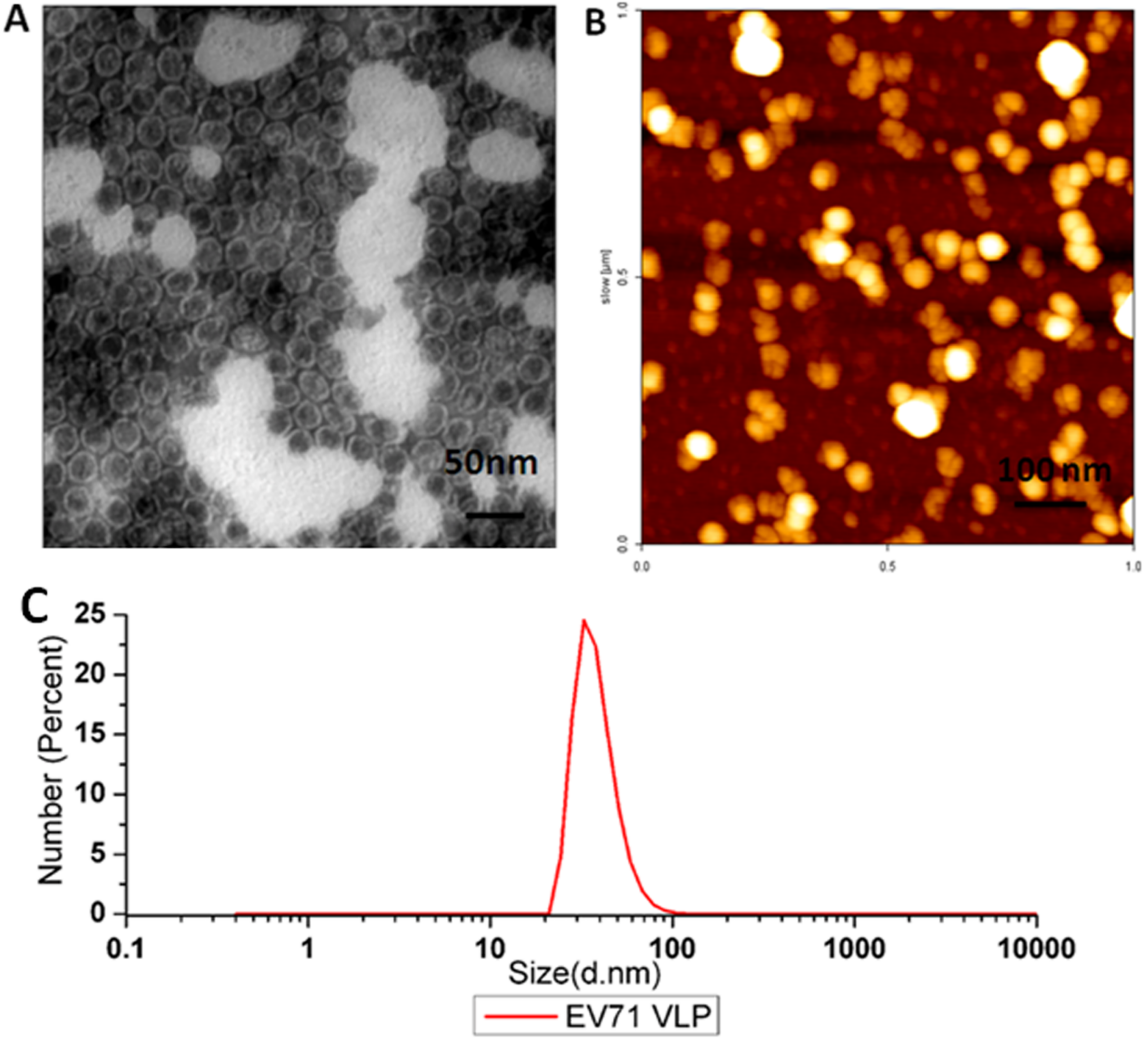
Morphology of EV71 VLPs. (A) TEM of purified EV71 VLPs negatively stained with 1% phosphotungstic acid (diameter ~30 nm), bar = 50 nm. (B) AFM image of purified EV71 VLPs (diameter ~57 nm), bar = 100 nm. (C) DLS of purified EV71 VLPs. The hydrodynamic diameter of EV71 VLPs was ~ 38 nm.

A representative TEM image is provided in Fig 4A. The average diameter as measured by TEM was approximately 30 nm for the purified EV71 VLPs, identical to the expected value for the native virus [24]. Moreover, Fig 4B shows an in-solution AFM image of our purified EV71 VLPs stabilized on a mica surface, which is believed to better preserve the native protein structure, as opposed to imaging on the dried sample, and maintain the particle height on the mica surface. Overall, the biophysical properties of EV71 VLPs, as estimated by the TEM and AFM images, suggested that the purified EV71 VLPs were empty capsids devoid of nucleic acid and retained good morphology. The Zetasizer Nano ZS instrument was used to test the hydrodynamic diameter and stability of EV71 antigen particles based on the uniformity, as VLPs have been reported to potentially aggregate or dissociate [32, 33]. The results suggested that the final purified VLPs were uniform with a diameter of approximately 38 nm (Fig 4C), similar to previous measurements of EV71 VLPs [32].

### Structural characterization of recombinant EV71VLPs

**Fig 5:**
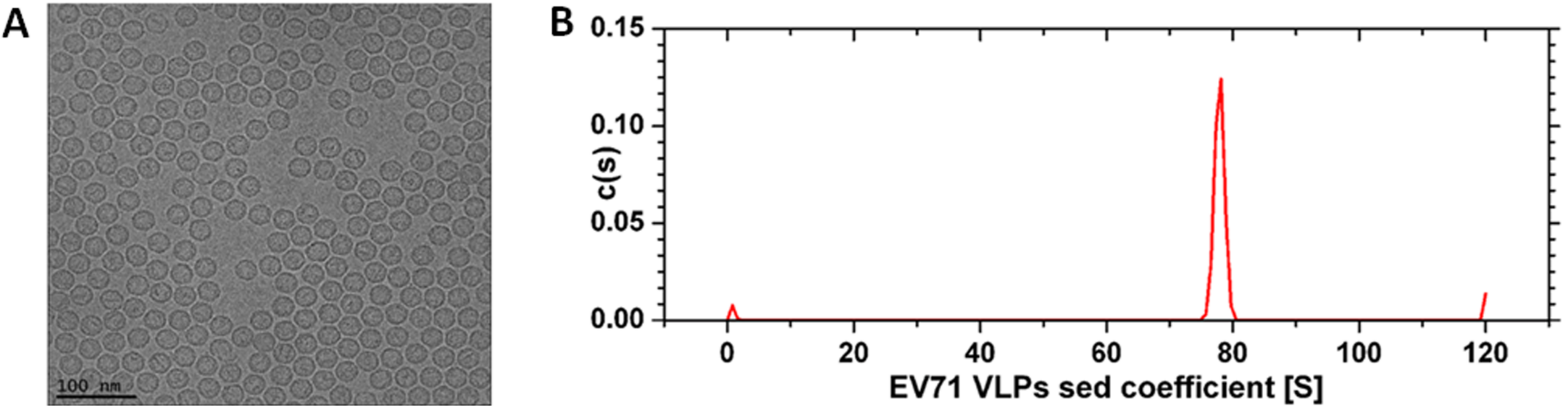
Characterization and cryo-TEM imaging of recombinant EV71 VLPs. EV71 VLPs purified by a multistep chromatographic process were characterized by cryo-TEM (A) and AUC (B).

Cryo-TEM, which allows for direct imaging of fully hydrated biological specimens in a near-physiological environment, revealed that the EV71 VLPs were 28.74 nm in diameter (Fig 5A), similar to the size of the authentic EV71 virus [24]. AUC analysis, which is a classical method for determining the molecular mass and size of proteins, suggested that the sedimentation coefficient of the EV71 VLPs was about 78S (Fig 5B), a result that is similar to previous measurements of native antigenic empty capsids of EV71 (~82S) which had an outer surface that was nearly identical to that of the native virion [24, 34]. The slight difference of sedimentation coefficients between EV71 VLPs (78S) and EV71 native virion (~82S) may be caused by the different degree of glycosylation of insect cells and mammalian cells. However, the sedimentation coefficient of the secreted form of EV71 VLPs (~85S) purified by CsCl density gradients [23] was greater than that of the purified EV71 VLPs in this study. As the difference in sedimentation coefficient between these two EV71 VLPs was actually quite substantial, some differences in structure may be present and should be further investigated. However, these results indeed demonstrated that highly purified EV71 VLPs were obtained with well-preserved structural characteristics of the naturally occurring empty EV71 capsid.

## Discussion

In this work, evidence is presented that EV71 VLPs of the C4 strain produced from the BEVS and purified through our multiple chromatography process retained key molecular and structural features for the first time, compared with those reported for native empty virions [24] and other EV71 VLPs [23, 35, 36]. The sedimentation coefficient of purified EV71 VLPs in this study (78S) was different from that of EV71 VLPs purified by CsCl (85S) as previously reported by Gong et al. [23] using AUC analysis, and SDS-PAGE results showed that our EV71 VLPs consisted of VP0 (35 KDa), VP1 (33 KDa) and VP3 (26 KDa) capsid proteins were also different from EV71 VLPs consisted mostly of VP0 (35KDa), VP1(33 and 30KDa), VP3(27kDa), and a presumably incompletely processed band VP0-VP3(62kDa) purified by Gong et al. As different strains, culture conditions, and storage conditions may influence on the final production[24, 35, 37], we speculate that these difference may be caused by our different production conditions. Although they were both BEVS-produced, the purified EV71 VLPs in this study were shown to be mainly located in the cytoplasm. The purity of the EV71 VLPs was characterized by SDS-PAGE, Western blotting, HPLC and DLS, and their sequences and structural profile were determined by amino acid analysis, mass spectroscopy, TEM, AFM and cryo-TEM. All of the results confirmed that our purified EV71 VLPs possessed similar characteristics to that described for the EV71 native empty capsid [24] with good molecular and structural properties.

Analysis of mass and amino acid sequences suggested that the amino acid sequences of the EV71 VLPs produced by BEVS were consistent with the native empty capsid at the hydroxyl- and carboxy-terminal ends. Moreover, no mutations were observed in the peptide mapping of VP0, VP1 and VP3 subunits of the purified EV71 VLPs. Since insect cells infected with recombinant baculoviruses can usefully express recombinant proteins, particularly when high production levels and post-translational modifications of the protein are desired [38], a determination of glycosylation site sequences of the EV71 VLPs produced from Sf9 cells was performed using MALDI-TOF/TOF, and an occupied N-glycosylation site was detected at VP1 residue N^176^. This direct investigation into the glycosylation of BEVS-produced EV71 VLPs was important, and as their expression in eukaryotic cells resulting in post-translational modifications may affect their folding, localization, solubility and antigenicity [39]. Protein glycosylation is regarded as one of the most important properties determining product quality towards affecting the physiochemical characteristics, for example, the secretion, stability, and immunogenicity[40, 41]. Mammalian, insect, plant, and yest host cell expression systems have been used for recombinant glycoproteins[42]. Even using the same cell line and culture media, the different culture conditions frequently result in different protein glycosylation[43]. Hyakumura et al.[44] have generated hepatitis B virus (HBV) VLPs with additional N-glycosylation sites on the envelope proteins (HBsAgS), and the result showed that the HBV VLPs with the highest N-glycan density can induce the enhanced immunogenicity, earlier and longer-lasting antibody immune responses than do wild-type. Given the importance of the glycosylation modification, our identified N-glycosylation site in EV71 VLPs at the VP1 residue N^176^ may need further study to verify whether the identified N-glycosylation site in EV71 VLPs at the VP1 residue N^176^ is essential. Using the site-directed mutagenesis, changing N^176^ into G^176^, we will test the effect of N-glycosylation site on the EV71 VLPs assembly used by TEM and AUC and HPLC, and on the humoral or cellular immune responses through immunization of BALB/c mice injected intraperitoneally.

Recombinant EV71 VLPs produced from yeast [35] and Sf9 cells [23] have been reported. However, those EV71 VLPs were purified by sucrose or CsCl density gradients. Given that different culture and purification methods may influence the production of recombinant VLPs, we sought to analyze potential differences in characteristics of our EV71 VLPs. In this study, membrane-like structures within the infected Sf9 cells were increased with time and were associated with apoptosis. The intracellular localization of our EV71 VLPs could be detected by immunofluorescence and immunogold staining. The results may provide a foundation for production of EV71 VLPs by determining their cytoplasmic localization and harvest time of Sf9 cells infected by recombinant baculovirus.

The purified EV71 VLPs, with a diameter of approximately 38 nm as analyzed by DLS (Fig 4C) and the morphological structure of empty particles observed by TEM (Fig 4A), were consistent with those obtained previously [13, 31]. These results suggested that the particles obtained in this study were structurally stable and similar to natural viruses. Analysis by DLS gives a hydrodynamic radius that corresponds to the core of the EV71 VLPs, whereas that obtained by TEM often gives the size of the core of EV71 VLPs in a dried state. As a consequence, the cryo-TEM analysis is necessary whenever a precise characterization of the structure of the EV71 VLPs is required. Here, the cryo-TEM measurement revealed a diameter of 28.74 nm for the EV71 VLPs (Fig 5A), which is similar to the size of the authentic EV71 virus. In addition to imaging, AFM provided a 3-dimensional profile of the surface of EV71 VLPs, which is needed to manipulate the molecules. The AFM height image showed that the diameter of the particles was approximately 57 nm. The larger value obtained from AFM compared to that from cryo-TEM may be caused by the radius of curvature. Nevertheless, these results revealed that the purified EV71 VLPs maintained good morphology and structure and were consistent with natural EV71 viruses.

The culture and purification methods may also influence the antigenicity and immunogenicity. Interestingly, although a difference between the sedimentation coefficient of our EV71 VLPs and that previously reported [23], these VLPs were similar to the empty capsid of natural EV71 virions. Therefore, these different EV71 VLPs should be further compared to identify whether they have differences in their structure. Moreover, our previous observation showed that 2^15^ neutralization antibody titer and 100% immune protection against EV71 homologous virus attacks in suckling mice could be elicited [22], supporting the conclusion that our EV71 VLPs had well-preserved molecular and structural characteristics.

Taken together, this study located the distribution of EV71 VLPs expression at the cellular level, and our results demonstrated that EV71 VLPs produced from the BEVS and prepared through a multi-step purification process retained good characteristics as a vaccine antigen. Because of the well-characterized EV71 VLPs antigen tested in this study, the EV71 VLPs prepared by us for vaccine antigen is expected to lay a foundation for the vaccine against HFMD research and development.
